# Removal of phosphate from River water using a new baffle plates electrochemical reactor

**DOI:** 10.1016/j.mex.2018.10.024

**Published:** 2018-10-31

**Authors:** Khalid S. Hashim, Ibijoke Adeola Idowu, Nisreen Jasim, Rafid Al Khaddar, Andy Shaw, David Phipps, P. Kot, Montserrat Ortoneda Pedrola, Ali W. Alattabi, Muhammad Abdulredha, Reham Alwash, K.H. Teng, Keyur H. Joshi, Mohammed Hashim Aljefery

**Affiliations:** a Department of Civil Engineering, Liverpool John Moores University, Liverpool, UK; b Department of Environment Engineering, University of Babylon, Babylon, Iraq; c Department of Environment Engineering, University of Wasit, Wasit, Iraq; d Department of Environment Engineering, University of Karbala, Iraq

**Keywords:** Electrocoagulation, Electrocoagulation, Phosphate, Multiple regression model, Hydrogen gas, Operating cost

## Abstract

During the last 50 years, the human activities have significantly altered the natural cycle of phosphate in this planet, causing phosphate to accumulate in the freshwater ecosystems of some countries to at least 75% greater than preindustrial levels, which indicates an urgent need to develop efficient phosphate treatment methods. Therefore, the current study investigates the removal of phosphate from river water using a new electrochemical cell (PBPR). This new cell utilises perforated baffle plates as a water mixer rather than magnetic stirrers that require power to work. This study investigates the influence of key operational parameters such as initial pH (ipH), current density (Ј), inter-electrode distance (ID), detention time (t) and initial phosphate concentration (IC) on the removal efficiency, and influence of the electrocoagulation process on the morphology of the surface of electrodes.

Overall, the results showed that the new reactor was efficient enough to reduce the concentration of phosphate to the permissible limits. Additionally, SEM images showed that the Al anode became rough and nonuniform due to the production of aluminium hydroxides. The main advantages of the electrocoagulation technique are:

• The EC method does not produce secondary pollutants as it does not required chemical additives, while other traditional treatment methods required either chemical or biological additives [[1], [2], [3], [4]].
• It has a large treatment capacity and a relatively short treatment time in comparison with other treatment methods, such as the biological methods [1,[5], [6], [7]].
• The EC method produces less sludge than traditional treatment traditional chemical and biological treatment methods [8,9].

EC technology, like any other treatment method, has some drawbacks that could limit its performance. For instance, it still has a clear deficiency in the variety of reactor design, and the electrodes should be periodically replaced as they dissolve into the solution due to the oxidation process [2,10].

## Graphical abstract

**Figure:**
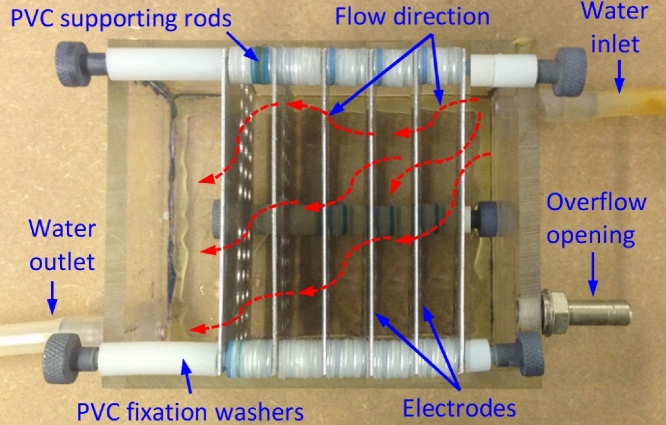

**Specifications Table**

**Table:** 

Subject area	*Environmental Science*
More specific subject area	*Water treatment*
Method name	*Electrocoagulation*
Name and reference of original method	Hashim et al. [2], Hashim et al. [8], and Hashim et al. [7].
Resource availability	

## Method details

### A. Reactor construction

**Fig. 1:**
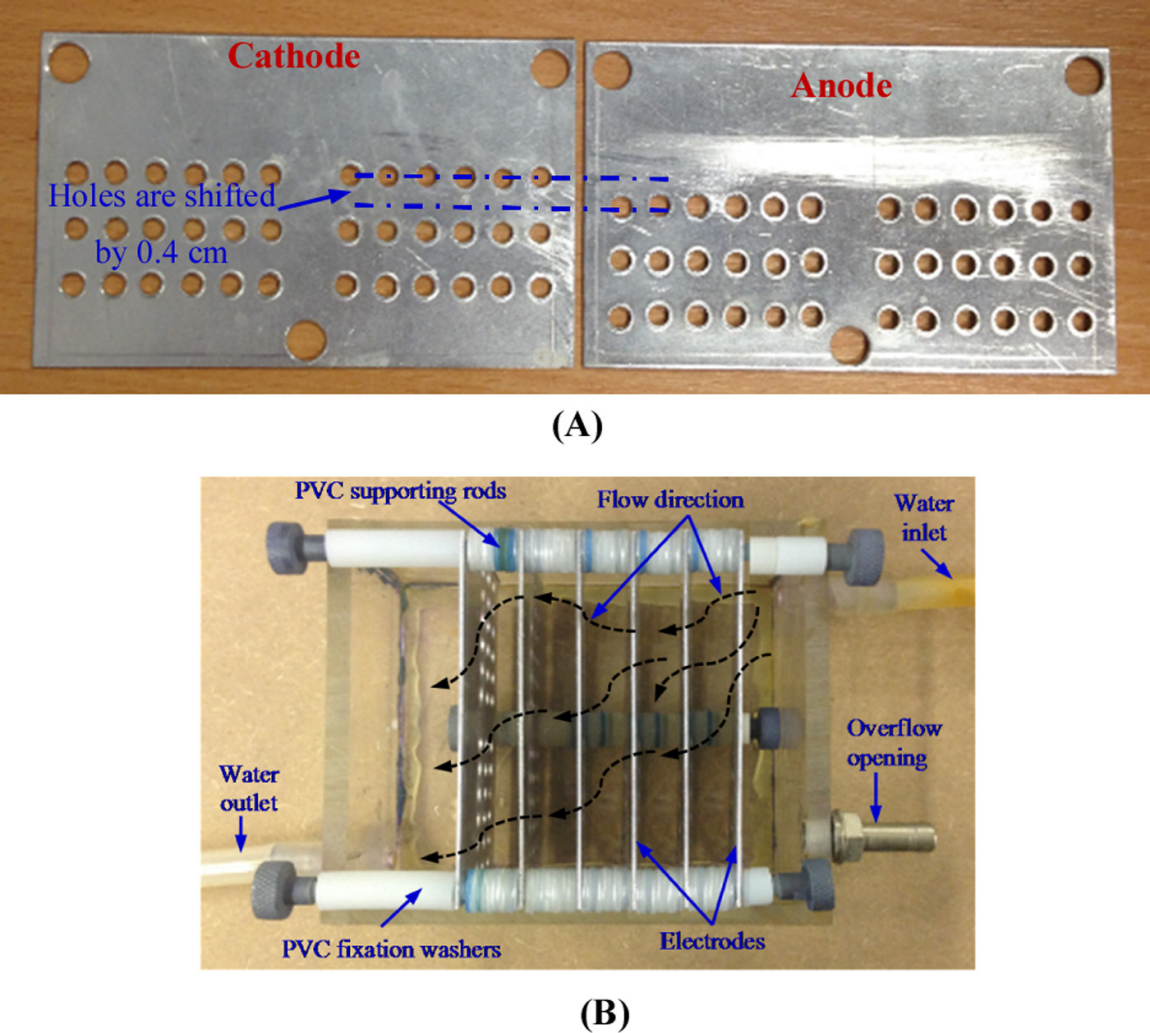
(A) Al electrodes, (B) The new electrocoagulation reactor (PBPR).

The electrochemical phosphates removal experiments have been carried out using a new rectangular electrocoagulation reactor (PBPR), as shown in Fig. 1. This reactor consists of a Perspex rectangular container of net dimensions of length 10 cm, width of 9.5 cm and a height of 7 cm. It is supplied with six parallel-perforated rectangular baffle plates (electrodes) made from aluminium. Each electrode, width of 9.4 cm and a height of 8 cm, has 36 holes (0.4 cm in diameter) distributed in three rows and three 0.7 cm diameter holes distributed at the top and bottom to fix it in the required position. It can be seen from Fig. 1(A) that the three rows of holes in the anode are shifted by 0.4 cm in comparison with those in the cathode, this is to ensure that the water follows in a convoluted path, thereby efficiently mixing the water being treated. The electrodes were held in the required position inside the reactor by 0.3 cm diameter PVC (Polyvinyl chloride) supporting rods. The distance between electrodes was controlled using 0.1 cm thickness PVC fixation washers. During the phosphate removal experiments, these electrodes were arranged in a monopole configuration and partially immersed in the water being treated (total effective area 304.4 cm^2^). The PBPR was connected to a peristaltic pump (Watson Marlow type, model: 504U) to circulate the water, and a rectifier (HQ Power; Model: PS 3010, 0–10 A, 0–30 V) to supply the required electrical current. Water temperature and pH values were measured using a pH/temperature pocket tester (Type: Hanna; Model: HI 98,130).

### B. Solutions

All chemicals used in the current investigation were supplied by Sigma-Aldrich and used as supplied. A stock phosphate synthetic solution, 100 mg P/L, was prepared by dissolving 439.4 mg of potassium diphosphate (${KH}_{2}{PO}_{4}$) per litre of deionised water. Samples of lower concentrations were prepared by dilution from this stock solution. The initial pH of the diluted samples was adjusted to the desired value using 1 M HCl or 1 M NaOH solutions, while water conductivity was modified using 6.5 mM of NaCl salt. All the runs were carried out at room temperature (20 ± 1 °C), which was controlled using a water bath (Nickel-Electro: Clifton).

The phosphate concentration was measured using standard Hach Lange phosphate cuvettes (LCK 348–350), according to the standard method provided, and a Hach Lange spectrophotometer (Model: DR 2800).

At the end of each experiment, the electrodes were removed from the reactor, cleaned with HCl acid and rinsed with deionised water before using them in the next experiment.

### C. Procedures and analysis

The electrochemical experiments were initiated by connecting the Al electrodes to the corresponding terminals of the rectifier. 500 mL of freshly prepared phosphate solution of the desired concentration, was fed into the PBPR and kept circulated, using the peristaltic pump, during the course of experiment. Treatment time was started when the rectifier was switched on.

Progress of phosphate removal was monitored by collecting 0.5 mL samples from the reactor at 5-minute intervals during the course of the experiment. The collected samples were filtered with 0.45 μm filters (Sigma-Aldrich) to separate the unwanted sludge. The filtrate was then labelled and refrigerated to be tested at the end of each experiment. The residual phosphate concentration was measured, as mentioned before, using a standard phosphate cuvette test. The removal efficiency (R%) was calculated using the following equation [1,4]:

(1) $$R\%=\;\frac{IC-FC}{IC}\times 100\%$$

where IC and FC are the initial and final concentrations of phosphate, in mg/L, respectively. Power consumption ($C_{power}$) was calculated using the following formula [11,12]:

(2) $$C_{power}=\;\frac{I*V*t}{Vol.}$$

where $C_{power}$ is the power consumption (W.h/m^3^), *I* is the applied current (A), *V* is the potential (V), *t* is the electrolysis time (hrs), and *Vol.* is the volume of solution (m^3^).

### D. Economic analysis

The operating costs include fixed costs such as construction and equipment costs, and running costs including the cost of energy, chemicals, sludge treatment, labour and maintenance [7,13].

However, the operating costs of a lab scale EC unit only comprise the cost of energy, chemicals, and electrode material [13,14]. In the current study therefore, the following equation has been used to calculate operating costs:

(3) $$Operating\;cost=\;\alpha \;C_{power}+\gamma \;C_{material}+\beta \;C_{chemicals}$$

where $C_{power}$ (kWh/m^3^), $C_{material}$ (kg Al/m^3^), and $C_{chemical}$ (kg /m^3^) are the consumed power, electrode material and chemicals, respectively. *α*, γ, and *β* are the unit prices of energy, electrode material and chemicals, respectively.

The amount of electrode material consumed during the electrolysing process is calculated using Faraday’s Law (Eq. (4)).

(4) $$C_{material}=\;\frac{I\;\times \;t\;\times \;m}{Z\;\times \;F}\;\times 10^{-3}$$

$C_{material}$ is the lost mass of the anode (kg), I the applied current (A), t the treatment time (second), m the molecular weight of electrode material (26.98 g/mol for Al), Z the number of electrons (3 for Al) and F Faraday’s constant (96487 C/mol).

### E. Statistical modelling of the removal process

The multiple regression technique (MRT) has recently gained increasing popularity as a modelling and/or optimising statistical tool due its ability to conduct complex investigations of the interrelationships among several variables [[15], [16], [17]]. Therefore, this technique has been used in the present investigation to develop an empirical model to reproduce the performance of PBPR in terms of phosphate removal.

## Method validation

**Fig. 2:**
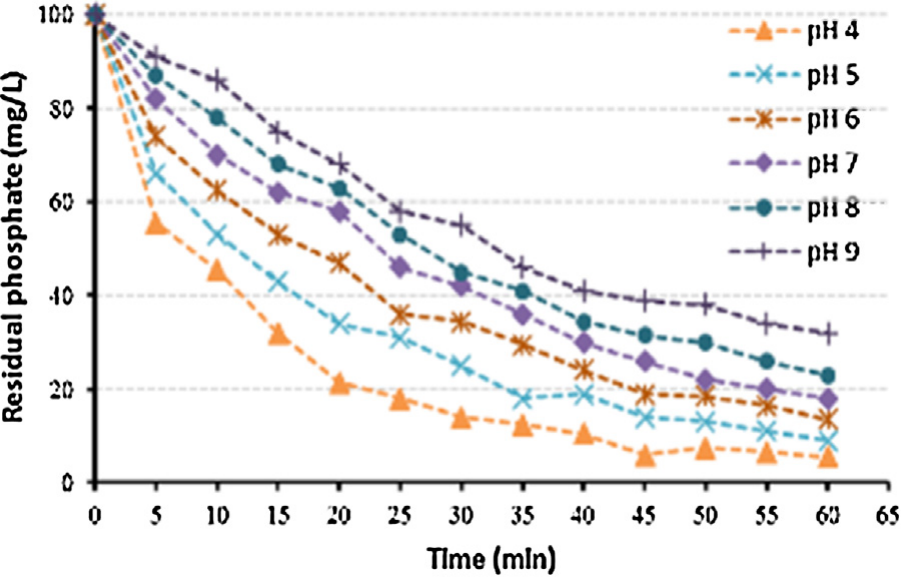
Phosphate removal efficiency versus treatment time for different initial pH values.

**Fig. 3:**
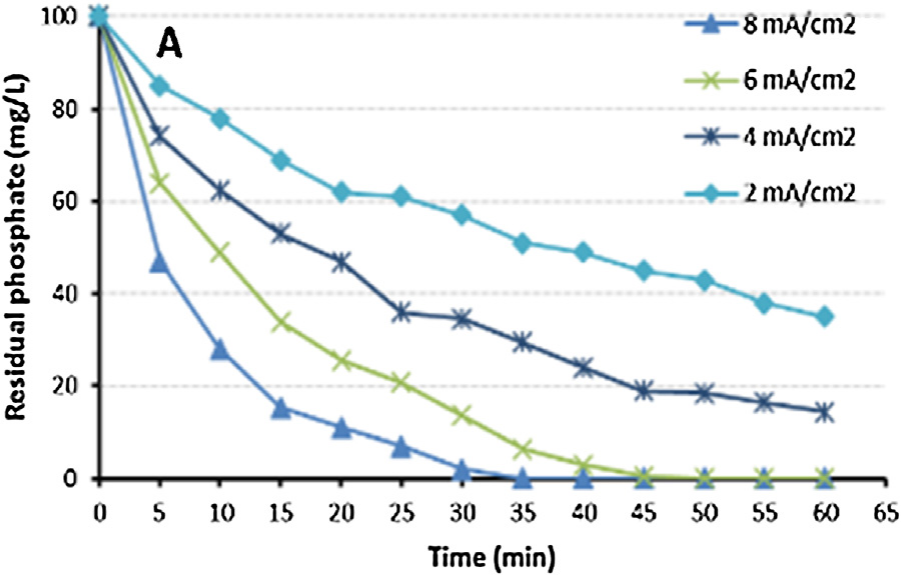
Influence of current density on phosphate removal.

**Fig. 4:**
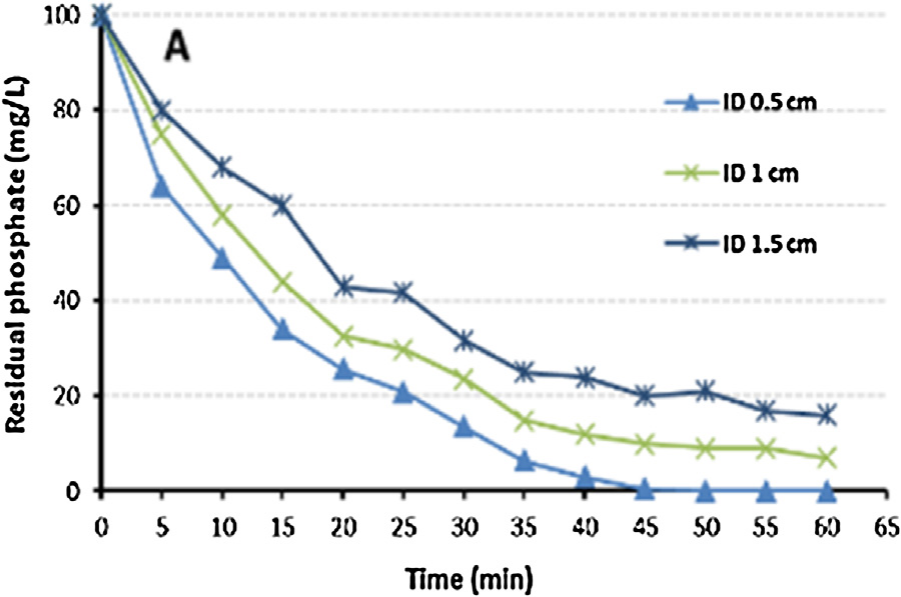
Influence of electrodes spacing on phosphate removal.

**Fig. 5:**
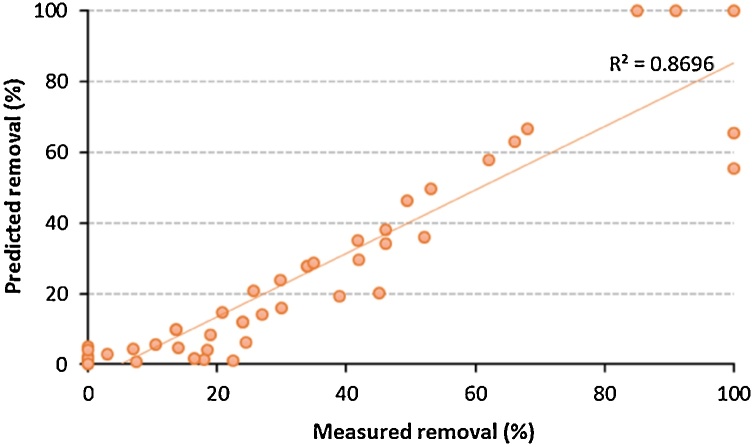
Measured versus predicted phosphate removal for randomly selected data points.

Fig. 2, Fig. 3, Fig. 4 describe the removal of phosphate as a function of different key operational parameters. The investigated ranges of these operating parameters, Table 1, were selected according to the literature [2,3,[6], [7], [8]]. Fig. 2, Fig. 3, Fig. 4, Fig. 5 show the influence of each single operational parameter on the removal of phosphate. Additionally, Fig. 5 shows a very good agreement between measured and predicted phosphate removal efficiencies (using the developed model).

**Table 1:** The investigated ranges of the studied operating parameters.

Parameter	Studied range	Unit
Initial pH	4–8	unitless
Current density	2–8	mA/cm^2^
Gap between electrodes	5–15	mm
Initial phosphate concentration	50–150	mg/L

This data indicated that the phosphate removal efficiency increased with the increase of current density, and decreased with the increase of gap between electrodes and the initial concentration of phosphate.
